# Correlation between antioxidant and anticancer activity and phenolic profile of new Apulian table grape genotypes (*V. Vinifera* L.)

**DOI:** 10.3389/fpls.2022.1064023

**Published:** 2023-01-11

**Authors:** Rosa Anna Milella, Mirko De Rosso, Marica Gasparro, Isabella Gigante, Giambattista Debiase, Lucia Rosaria Forleo, Antonio Domenico Marsico, Rocco Perniola, Valeria Tutino, Maria Notarnicola, Riccardo Velasco, Riccardo Flamini

**Affiliations:** ^1^ Research Centre for Viticulture and Enology, Council for Agricultural Research and Economics, Turi, Bari, Italy; ^2^ Research Centre for Viticulture and Enology, Council for Agricultural Research and Economics, Conegliano, Treviso, Italy; ^3^ Laboratory of Nutritional Biochemistry, National Institute of Gastroenterology Saverio de Bellis, IRCCS Research Hospital, Castellana Grotte, Italy

**Keywords:** breeding, phenolic compounds, seedless grape, antioxidant, anticancer, quality improvement

## Abstract

Grapes represent a significant source of phenolic compounds known for their health-promoting properties, such as antioxidant capacity on normal cells and prooxidant activity on tumor cells. The genotype highly affects the polyphenolic composition in grapes and, consequently, the nutritional quality of berries. This work aimed to characterize the phenolic composition, the antioxidant, and anticancer activity of grape skin extracts (GSEs) of nine new table grape genotypes selected from a breeding program to obtain new cultivars of seedless table grapes, well adapted to the climatic change and with higher nutraceutical properties. The grape polyphenolic profile was characterized by Ultra-High-Performance Liquid Chromatography/Quadrupole-Time of Flight mass spectrometry analysis. GSE antioxidant activity was determined by the ABTS, DPPH, and ORAC assays; GSE cell growth inhibition test was carried out in the Caco2 human cancer cell line. The nine GSEs showed different flavonoid and non-flavonoid profiles, and all possessed antioxidant activity, with the ‘Aika N.’, ‘Turese N.’, and ‘Egnatia N.’ the most active. As anticancer activity against the tested cancer cell line, ‘Daunia N.’ and ‘Apenestae N.’ showed the EC50 after 24 h of 35.60 µg/mL and 150.91 µg/mL, respectively. The relationship between polyphenolic profile and the antioxidant and anticancer activity of GSE was also investigated. Interestingly, among the different classes of polyphenolics, flavan-3-ols e proanthocyanidins showed the highest positive correlation with the anticancer activity of extracts. These findings can be helpful for the preparation of new extracts for the pharmaceutical and nutraceutical industry and geneticists working in vine breeding programs.

## 1 Introduction

Grapevine, *Vitis vinifera* subsp. *sativa* L., is one of the major fruit crops worldwide in economic value and cultivated area. Italy is the first European table grape producer, with 1.1 million tonnes of table grapes harvested in 2018 (OIV, 2019), mainly produced in the Apulia region, in the south of Italy. The demand for seedless grapes has increased considerably in the last few years. So along with “Italian historical varieties” such as ‘Victoria B.’, ‘Michele Palieri N.’, ‘Italia B.’, and ‘Red Globe RS.’, new seedless grape varieties from breeding programs are emerging to satisfy market demands. To date, breeding programs are essentially aimed at improving pathogen resistance and adaptation to climate change without neglecting berries’ nutritional quality, considering the increasing consumer awareness of the relationship between food consumption and health benefits.

Grape is one of the main natural dietary sources of polyphenols, secondary plant metabolites of great interest for nutritional and medical purposes. These bioactive molecules are associated with health-promoting properties such as antioxidant, antiatherogenic, antithrombotic, antimicrobial, anti-inflammatory activities, and protective effects against non-communicable diseases, including cancer and cardiovascular, metabolic, and neurodegenerative disorders (Câmara et al., 2020; Caponio et al., 2022). There are three main classes of polyphenols in grapes: flavonoids, phenolic acids, and stilbenes. Flavonoids share a typical basic structure (C6-C3-C6) with two benzene rings (Rings A and B) connected by an oxygenated heterocycle (Ring C). Based on the hydroxyl groups and variations in Ring C, flavonoids can be classified into six classes: flavonols, flavones, isoflavones, flavanols, flavanones, and anthocyanidins (Pietta et al., 2003). Non-flavonoid compounds in grapes include stilbene derivatives (C6-C2-C6), such as trans-resveratrol, cis and trans piceids, and several resveratrol dimers (viniferins), trimers, and tetramers (Flamini et al., 2013), and phenolic acid that exists mainly as hydroxybenzoic (C6-C1) and hydroxycinnamic acids (C6-C3), in either the free or conjugated form (Teixeira et al., 2013). The chemical structure makes grape polyphenols able to exert health benefits *via* multitarget mechanisms, interacting with enzyme systems involved in crucial pathways.

Among their abilities, grape polyphenols are excellent antioxidants capable of being potent scavengers of reactive oxygen/nitrogen species (ROS/RNS) and metal chelating agents, preventing more ROS generation, biomolecules damage, and performing a preventative strategy against mutation-related diseases (Arau´jo Vieira do Carmo et al., 2018). Many studies evaluated the antioxidant activities possessed by skins, seeds, stems, and pomace of grape berries by different methods (Lingua et al., 2016; Liu et al., 2018; Samoticha et al., 2018; Esparza et al., 2021; Negro et al., 2021; Qin et al., 2022), but only a few compared a large number of grape varieties (Liu et al., 2018; Sochorova et al., 2020). Beyond their antioxidant capacity on normal cells, they can act as a prooxidant on tumor cells resulting in potential antiproliferative agents. Some studies on the anticancer properties of table grape extracts have shown their cytostatic and cytotoxic effects on cancer cells (Kaur et al., 2008; Dinicola et al., 2012; Valenzuela et al., 2018). Our previous studies showed that table grape skin extracts of ‘Egnatia N.’ and ‘Autumn royal N.’ modulated cell proliferation and apoptosis in the Caco2 cell line (Gigante et al., 2020), blocked cell migration, exerted morphological changes on the cultured cells (Tutino et al., 2020a), influenced membrane fluidity by affecting its content of PUFAs (Tutino et al., 2020b).

Anticancer and antioxidant activities of phenolic compounds greatly depend on their chemical structures (Arau´jo Vieira do Carmo et al., 2018; Wang et al., 2018). Consequently, the phenolic composition of grapes, which is affected by the genotype, cultural practices, and environmental conditions (Xia et al., 2010), is crucial for determining health-promoting effects. Studies focused on table grape bioactive composition, and their health effects are very limited to date. Our study aimed to evaluate the phenolic composition, *in vitro* antioxidant, and antiproliferative activities of nine new table grape genotypes (‘Aika N.’, ‘Apenestae N.’, ‘Appia N.’, ‘Daunia N.’, ‘Egnatia N.’, ‘Maula N.’, ‘Murex N.’, ‘Netium N.’, ‘Turese N.’) selected from a breeding program carried out at the CREA-Research Centre for Viticulture and Enology of Turi (Italy). Furthermore, the relationship between the phenolic composition, antioxidant and anticancer activity was investigated.

## 2 Materials and methods

### 2.1 Plant material

**Figure 1:**
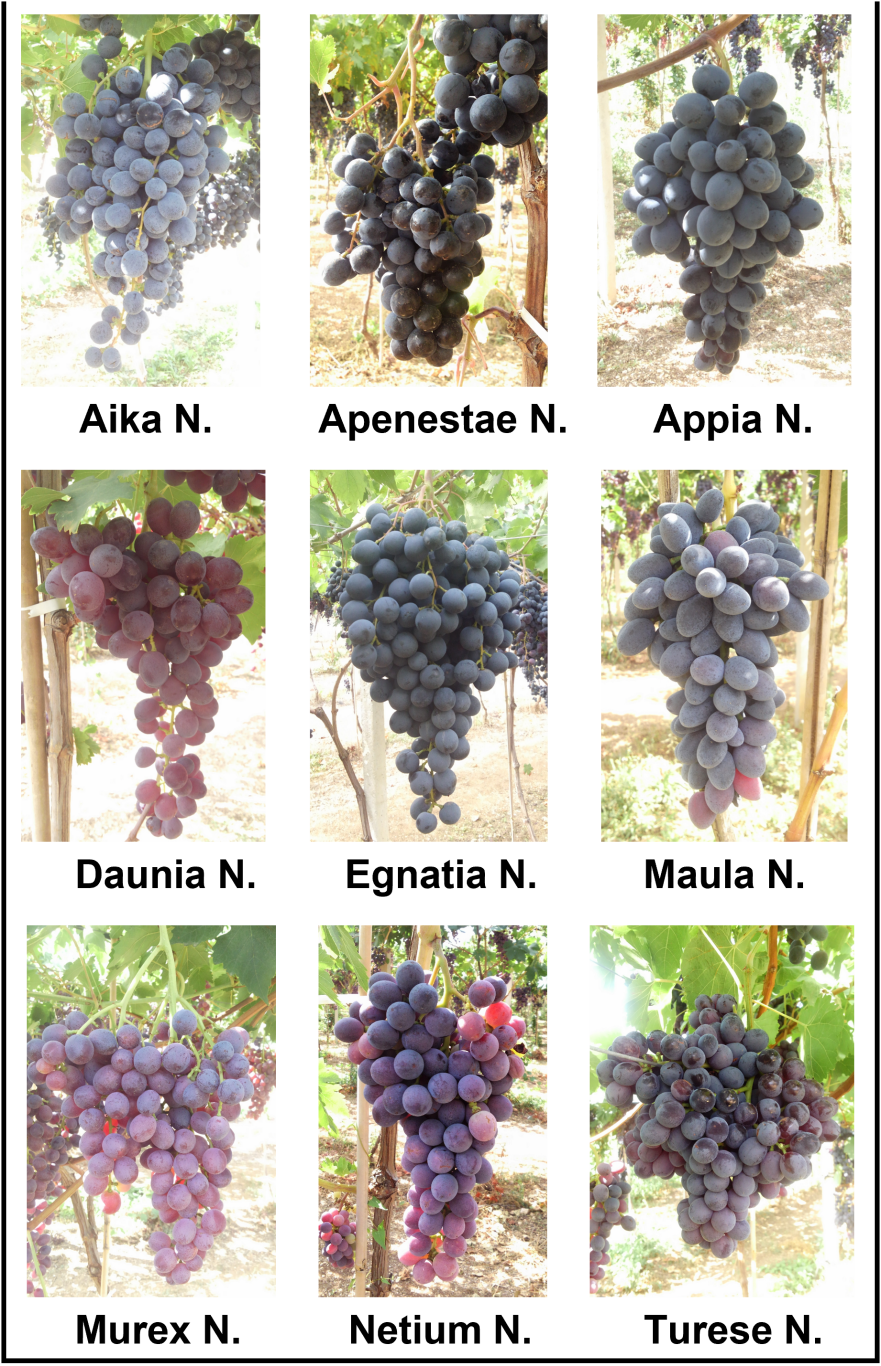
Nine new seedless grape genotypes, ‘Aika N.’, ‘Apenestae N.’, ‘Appia N.’, ‘Daunia N.’, ‘Egnatia N.’, ‘Maula N.’, ‘Murex N.’, ‘Netium N.’, ‘Turese N.’, selected from a breeding program carried at the CREA-Research Centre for Viticulture and Enology of Turi (BA).

Nine new seedless grape genotypes, ‘Aika N.’, ‘Apenestae N.’, ‘Appia N.’, ‘Daunia N.’, ‘Egnatia N.’, ‘Maula N.’, ‘Murex N.’, ‘Netium N.’, ‘Turese N.’ (**Figure 1**), were selected for this study from a breeding program (NUVAUT) carried out for more than ten years at the CREA-Research Centre for Viticulture and Enology of Turi. The program aimed at obtaining new cultivars of table grapes well locally adapted to the climatic conditions, seedless, and with higher nutraceutical properties. These new genotypes were obtained by crossing seeded varieties (as a female parent) with seedless varieties (as a male parent). In particular, the following crossing combination are involved: ‘Red Globe RS.’ x ‘Autumn royal N.’ (‘Aika N.’, ‘Apenestae N.’, ‘Appia N.’, ‘Egantia N.’, and ‘Netium N.’), ‘Red Globe RS.’ x ‘Regal B.’ (‘Daunia N.’, ‘Murex N.’ and ‘Turese N.’) and ‘Perla di Yalova B.’ x ‘Autumn royal N.’ (‘Maula N.’). All plants were grown at the same agricultural and environmental conditions in an experimental vineyard of CREA, located in Rutigliano (Southern Italy; 40°57’26” N; 17°00’26” E). Grapes were harvested in 2019 at technological maturity from five plants. For each genotype, six clusters were collected, and about 100 berries were picked randomly from different parts of the clusters and immediately frozen at -20°C.

### 2.2 Grape skin extract (GSE) preparation

For sample preparation, the skin from 30 berries was manually separated from the pulp, weighed, dried at 37°C, and ground with a blender until a fine powder. 0,25 g of powder was resuspended with 5 mL of ethanol solution: water: hydrogen chloride 37% (70:30:1 v/v/v). After 24 hours under dark conditions, the mixture was centrifuged at 4000g for 3 min, and the clear supernatant was filtered through a 0.2 µm syringe cellulose filter and immediately analyzed or stored at -20°C. For the cell proliferation assay, the supernatant was evaporated by a vacuum concentrator at room temperature until the volume of the liquid was less than 30% of the origin volume. All samples were brought to the same final volume. Before cell assay, the sample was filtered through a 0.2 µm syringe cellulose filter. All the prepared samples were kept at -20°C until further analysis.

### 2.3 Determination of total phenolic content (TPC), total anthocyanins (TA), and total flavonoids (TF)

TPC was determined using the microscale protocol described by Waterhouse (2009). Briefly, 1 mL of water, 0.02 mL of extract sample, 0.2 mL of the Folin-Ciocalteu reagent, and 0.8 mL of 10% sodium carbonate solution were mixed and brought to 3 mL. The absorbance was measured at 765nm after 90 min at room temperature with a spectrophotometer Agilent 8453 (Agilent Technologies, Santa Clara, CA). Results were expressed as milligrams of gallic acid equivalent/g of dry weight based on a gallic acid calibration curve (50 to 500 mg/L with R^2^ = 0.998).

The determination of TA was performed using a pH-differential protocol as proposed by Lee et al. (2005). Appropriate dilutions of grape extracts were mixed either in 0.025M potassium chloride (pH 1) or 0.4M sodium acetate (pH 4.5) buffers. Absorbance was measured at 520 and 700nm with the spectrophotometer system Agilent 8453 (Agilent Technologies, Santa Clara, CA). Results were expressed as milligram cyanidin 3-glucoside equivalents per gram of grape skin (mg Cy/g skin).

The TF was determined by the aluminum chloride method (Ayoola et al., 2008) with some modifications. 1 mL of the GSE (diluted 1:10 with ethanol) was mixed with 1 mL of 2% aluminum chloride and incubated at 25°C for 40 min. Then, the absorbance of the mixture was subsequently measured at 402 nm. The TF was calculated as mg of rutin equivalent per g (mg RE/g dw) of weight of samples using the calibration curve of quercetin (0–150 µg/mL).

### 2.4 Antioxidant activity

The antioxidant activity was evaluated by radical scavenging assays based on two different mechanisms: hydrogen atom transfer (HAT) and single electron transfer (SET). Precisely, two single-electron-transfer assays (ABTS radical cation and DPPH assays) and one hydrogen-atom-transfer-based assay (ORAC) were performed. Briefly, the DPPH (2,2 O-diphenyl-1-picrylhydrazyl) assay was conducted according to Brand-Williams et al. (1995) technique with some modifications. The stock solution was prepared by mixing 2.5 mg DPPH radical with 100 mL ethanol. The solution absorbance was adjusted at 0.7 ± 0.02 in 515 nm using a UV–Vis spectrophotometer Agilent 8453 (Agilent Technologies, Santa Clara, CA). 2 mL of DPPH radicals were placed in a test tube and 200 µL of the sample extract or standard were added (ethanol was used as blank). The decrease in absorbance at 515 nm was measured after 30 min of incubation at 37°C. Calibration curves were prepared using Trolox. DPPH values were expressed as µM Trolox equivalents/g of skin.

The ABTS (2,2-Azino-bis-3-ethyl benzotiazoline-6-sulfonic acid) assay was conducted according to Miller et al. (1993) with some modifications. ABTS^+^ cation was generated by mixing 0.5mM ABTS dissolved in pH 4.5 acetate buffer with 25 mM potassium persulfate, a ratio of 200/1 v/v. ABTS^+^ was incubated in the dark at room temperature for 16 h. Successively, the activated radical was diluted with acetate buffer to an absorbance of 0.70 ± 0.02 at 734 nm. 200 µL of sample or standard was added to 2000 µL of diluted ABTS solution; absorbances were recorded after 20 min of incubation at 30°C. Calibration curves were prepared using Trolox, and results are expressed as µM Trolox equivalents/g of skin.

Oxygen Radical Absorbance Capacity (ORAC) assay was performed as described in Gillespie et al., 2007. Briefly, ORAC analysis was carried out using a plate reader FLUOstar OPTIMA (B.M.G. Labtech, Germany), fluorescein as a probe with an excitation wavelength of 485 nm, and an emission wavelength of 520nm. ORAC values were expressed as µM Trolox equivalents/g of skin.

The Antioxidant Composite Index (ACI) was calculated to provide comprehensive information about the antioxidant activity of extracts. Combining the results from all performed tests (DPPH, ABTS, and ORAC) enabled the ACI calculation. An Antioxidant Index Score were computed by assigning a score of 100 to the highest value in each assay and subsequently calculating the score for all other samples as a percentage of the highest score following the equation:


[1] $$\text{Antioxidant Index Score}=\frac{\text{sample score}}{\text{best score}}\times 100$$

The ACI was the average of the three antioxidant activity indices for each grape genotype (Seeram et al., 2008; Dabetić et al., 2020).

### 2.5 Human colon cancer cell culture and treatment

Human colon adenocarcinoma-derived Caco2 cell line (well‐differentiated) (G1–2) (from adenocarcinoma) were purchased from the American Type Culture Collection (ATCC) Cell Bank (Manassas, Virginia). Cells were grown in Roswell Park Memorial Institute (RPMI) 1640 medium, supplemented with 10% fetal bovine serum (FBS), 100 U/mL penicillin, and 100 μg/mL streptomycin, 2 mM glutamine, in monolayer cultures, and incubated at 37°C in a humidified atmosphere containing 5% CO_2_ in the air. For the experimental group, the nine GSEs (‘Aika N.’, ‘Apenestae N.’, ‘Appia N.’, ‘Daunia N.’, ‘Egnazia N.’, ‘Maula N.’, ‘Murex N.’, ‘Netium N.’ and ‘Turese N.’) were added to the medium at increasing concentrations, and the control group received the same volume of treatment in the solvent of GSEs (no treatment); the cells were then incubated at 37°C in an atmosphere of 5% CO_2_ in the air, for the experimental times indicated below.

### 2.6 MTT assay

Cells were cultured for 24, and 48 h with increasing concentrations of nine grape variety derived GSEs (20, 40, and 80 μg/mL) dissolved in ethanol:water:hydrogen chloride 37% (70:30:1 v/v/v), and no treatment for the control group. The effect on cell proliferation was analyzed by performing a colorimetric 3‐(4,5-dimethylthiazol‐2‐yl)‐2,5‐diphenyltetrazoliumbromide (MTT) assay (Sigma Aldrich; Milan, Italy), as previously described (Gigante et al., 2020; Tutino et al., 2020a). The median effective dose (EC50) means the concentration of GSE inhibits cell proliferation by 50%. The EC50 was calculated by GraphPad Prism 9 and expressed as µg of GSE per milliliter of cell culture (µg GSE/mL).

### 2.7 UHPLC/QTOF mass spectrometry

Two milliliters of the extract were 3-fold diluted using H_2_O/CH_3_CN 95:5 (v/v), a volume of 100 μL of 4’,5,7-trihydroxy flavanone 100 mg/L solution was added as internal standard (IS). The analysis was performed using a system composed by Ultra-High Performance Liquid Chromatography (UHPLC) Agilent 1290 Infinity coupled to Agilent 1290 Infinity Autosampler (G4226A) and Agilent 6540 accurate-mass Quadrupole-Time of Flight (QTOF) Mass Spectrometer (nominal resolution 40.000) with Dual Agilent Jet Stream Ionization source (Agilent Technologies, Santa Clara, CA). Analysis of each sample were performed in both positive and negative ionization modes, two analytical repetitions each were performed, and data were recorded in full scan acquisition mode. Chromatographic separation was performed using a Zorbax reverse-phase column (RRHD SB-C18 3×150 mm, 1.8 µm) (Agilent Technologies, Santa Clara, CA) and mobile phase composed by A) 0.1% v/v aqueous formic acid, and B) 0.1% v/v formic acid in acetonitrile. Gradient elution program: 5% B isocratic for 8 min, from 5% to 45% B in 10 min, from 45% to 65% B in 5 min, from 65% to 90% in 4 min, 90% B isocratic for 10 min. Flow rate 0.4 mL/min; sample injection: 10 µL; column temperature: 35°C. To check false positives after each sample a blank composed by mixture of the two mobile phases 1:1 v/v, was analyzed.

QTOF conditions: sheath gas nitrogen 10 l/min at 400°C; dehydration gas 8 L/min at 350°C; nebulizer pressure 60 psi; nozzle voltage 0 kV (negative mode) and 1 kV (positive mode); capillary voltage ±3.5 kV in positive and negative ion modes, respectively. Signals were recorded in the *m/z* 100–1700 range at acquisition rate 2 spectra/s. Mass calibrations were performed using standard mix G1969-85000 (Supelco Inc.), residual error for the expected masses ±0.2 ppm. Negative ionization lock masses: TFA anion at *m/z* 112.9856 and HP-0921 at *m/z* 966.0007 (ion [M^+^HCOO]^−^); positive ionization lock masses: purine at *m/z* 121.0509 and HP-0921 at *m/z* 922.0098. MS/MS fragmentation of parent ions selected in the *m/z* 100-1700 range was performed using collision energies between 20-60 eV. Acquisition rate 2 spectra/s.

MS/MS fragmentation of the precursor ions selected in the *m/z* 100-1700 range using collision energies between 20-60 eV, were performed. Spectra were recorded at acquisition rate 2 spectra/s. Compounds annotation were assigned on the basis of the spectral features (maximum mass error ±2 ppm), the chromatographic sequence elution and using the data collected in the homemade database *GrapeMetabolomics* (Flamini et al., 2013). Study of the samples was performed using the metabolites identified at levels 1, 2a, and 2b of Metabolomics Standards Initiative (MSI) (level 1 - using the standard compound; level 2a - agreement between MS/MS fragments and mass spectral information found the literature and online spectra databases; level 2b - structure tentatively assigned on the MS/MS fragments (Sumner et al., 2007).

Standards of quercetin, quercetin-3-*O*-glucoside, isorhamnetin-3-*O*-glucoside, kaempferol-3-*O*-glucoside, rutin, procyanidin B1, procyanidin B2, (+)-catechin, (−)-epicatechin, and (−)-epicatechin-3-*O*-gallate were purchased from Extrasynthese (Genay, France); *trans*-resveratrol (3,5,4’-trihydroxystilbene), piceatannol (3,4,3’,5’-tetrahydroxy-trans-stilbene), *E*-piceid, and 4’,5,7-trihydroxy flavanone were purchased from Sigma-Aldrich (Milan, Italy); δ-viniferin was provided by CT Chrom (Marly, Switzerland). *Z*-piceid produced by isomerization of *E*-isomer and *E*-ϵ-viniferin extracted from vine cane as previously reported, were used (Flamini et al., 2013).

Data acquisition software was Agilent MassHunter version B.04.00 (B4033.2). Data analysis was performed using Agilent MassHunter Qualitative Analysis software B.05.00 (5.0.519.0).

### 2.8 Statistical analysis

Data were analyzed by STATGRAPHICS Centurion software (Stat-Ease, Minneapolis, MN, USA). Results are expressed as mean ± SD (n = 3 for the instrumental data). After verifying the normal distribution (Shapiro-Wilks test, p>0.05) and the homoscedasticity (Levene’s test, p>0.05) of data significant differences among triplicate of assays were assessed using one-way ANOVA. Tukey’s *post hoc* test was used to compare the means (p<0.05). The antiproliferative effects were determined by Paired T-test. Data were expressed as mean ± standard deviation. The correlation between phenolics, antioxidant properties, and antiproliferative activities was assessed by calculating Pearson’s coefficient r and statistical reliability. The correlation was considered very strong when r = 0.90-0.99 (positive) or -0.99-(-0.99) (negative); strong when r = 0.70-0.89 (positive) or -0.89-(-0.70) (negative); and moderate when r = 0.40-0.69 (positive) or -0.69-(-0.40) (negative).

## 3 Results

### 3.1 Total phenolic content (TPC), total flavonoids (TF), and total anthocyanins (TA)

The TPC, TA, and TF of nine new Apulian table grape genotypes were reported in **Table 1**. Estimation of total phenolic content (TPC) using Folin–Ciocalteu reagent is widely adopted to study natural antioxidants, measuring the capacity of a compound to react with specific redox complex Folin-Ciocalteu reagent to form a blue complex that can be quantified by visible-light spectrophotometry at 765 nm. The TPC of GSEs was significantly different among the new genotypes, ranging from 23.26 to 39.65 mg GAE/g DM. In particular, ‘Appia N.’ had the highest value of TPC (39.65 mgGAE/g DM), followed by ‘Egnatia N.’ and ‘Aika N.’ (37.45 and 34.45 mgGAE/g DM, respectively). The lowest values appeared in ‘Maula N.’, ‘Murex N.’, ‘Netium N.’, and ‘Daunia N.’ (23.26, 25.65, 27.39, 27.46 mgGAE/g DM). The spectrophotometric assay for total flavonoid content revealed slight differences among all the new grape genotypes analyzed, with ‘Turese N.’ and ‘Appia N.’ with the highest value (3.87 and 3.69 mgRE/g DM) and ‘Murex N.’ with the lowest (1.70 mgRE/g DM). Regarding the total anthocyanins (TA) in the GSEs, data showed that ‘Egnatia N.’ was the richest (12.65 mg CyE/g DM), followed by ‘Turese N.’ and ‘Aika N.’ (11.84 and 10.94 mg CyE/g DM). In contrast, ‘Daunia N.’, ‘Murex N.’, and ‘Netium N.’ possessed the lowest contents (4.07, 4.21, 4.96 mg CyE/g DM, respectively).

**Table 1:** Total phenolic content (TPC), total anthocyanins (TA), total flavonoids (TF), antioxidant activity (ABTS, DPPH, and ORAC assays) and Antioxidant Composite Index (ACI) of nine new Apulian genotypes.

Genotype	TPC	TA	TF	ABTS	DPPH	ORAC	ACI
	(mg GAE/g)	(mg CyE/g)	(mg RE/g)	(µM TE/g)	(µM TE/g)	(µM TE/g)	%
Aika N.	34.45 ± 3.40abc	10.94 ± 0.50b	2.95 ± 1.47abc	481.1 5 ± 34.59ab	116.62 ± 16.01bcd	601.51 ± 50.20a	82.74 ± 26.12
Apenestae N.	33.07 ± 3.00bc	8.27 ± 0.72d	3.00 ± 0.52abc	414.44 ± 48.47bc	122.06 ± 13.77bcd	552.82 ± 141.22ab	76.45 ± 19.06
Appia N.	39.65 ± 1.07a	9.50 ± 0.09c	3.69 ± 0.27a	503.62 ± 45.83a	140.59 ± 5.27bc	549.44 ± 19.31ab	84.96 ± 19.06
Daunia N.	27.46 ± 1.17de	4.07 ± 0.12e	1.92 ± 0.03bc	329.62 ± 11.26d	100.61 ± 5.73bcde	407.83 ± 21.07bc	59.57 ± 12.28
Egnatia N.	37.45 ± 0.74ab	12.65 ± 0.14a	3.38 ± 0.21ab	468.77 ± 11.04ab	221.32 ± 35.73a	565.53 ± 44.38ab	95.70 ± 3.75
Maula N.	23.26 ± 1.57e	8.74 ± 0.12cd	2.49 ± 0.25abc	302.21 ± 4.18d	65.17 ± 9.06e	349.45 ± 14.30c	49.18 ± 17.12
Murex N.	25.65 ± 0.62de	4.21 ± 0.03e	1.70 ± 0.15c	344.78 ± 22.76cd	145.34 ± 21.06b	378.12 ± 36.09c	65.67 ± 2.80
Netium N.	27.39 ± 0.52de	4.96 ± 0.12e	2.04 ± 0.36bc	346,00 ± 20.96cd	96.6 ± 3.22cde	379.14 ± 54.68c	58.46 ± 13.14
Turese N.	29.93 ± 1.93cd	11.84 ± 0.24ab	3.87 ± 0.47a	434.96 ± 4.41ab	91.93 ± 1.12de	594.99 ± 18.51a	75.61 ± 30.16

In each column, values followed by different letters are statistically different according to the Tukey’s *post hoc* test (*p* < 0.05).

### 3.2 Antioxidant activity

In this study, the antioxidant activity of GSEs was evaluated by two different single-electron-transfer assays (ABTS radical cation and DPPH assays) and one hydrogen-atom-transfer-based assay (ORAC assay) (**Table 1**). The three different antioxidant assays revealed similar trends for all genotypes showing the ‘Aika N.’, ‘Turese N.’, and ‘Egnatia N.’ as the most active table grapes and ‘Maula N.’ as the least effective. In detail, **Table 1** reported the results of the three antioxidant activity tests.

The ABTS assay is based on the ability of grape skin antioxidants to reduce the ABTS green–blue cation radical through an electron transfer mechanism visualized as a discoloration. ‘Appia N.’ (503.62 µmol TE/g DM), followed by ‘Aika N.’, ‘Egnatia N.’, and ‘Turese N.’ (481.15, 468.77, and 434.96 µmol TE/g DM, respectively) showed the highest ABTS value. On the contrary, ‘Maula N.’ possessed the lowest ABTS value (302.21 µmol TE/g DM).

In the DPPH assay, the grape skin antioxidants react with a stable free radical DPPH•(2,2-diphenyl-1-picrylhydrazyl, λ_max_=517 nm), causing discoloration of the molecule. ‘Egnatia N.’ had the highest DPPH value and was significantly higher than other cultivars. ‘Maula N.’ possessed the lowest antioxidant activity.

The ORAC assay measures the degree of inhibition of peroxy-radical-induced oxidation by grape phenolic compounds based on the hydrogen atom transfer reaction. ‘Aika N.’ and ‘Turese N.’ possessed the higher values (601.51 and 594.99 µmol TE/g), while ‘Maula N.’, ‘Murex N.’, and ‘Netium N.’ showed the lowest ORAC values (349.45, 378.12, and 379.14, respectively). Compared to the others, this assay found fewer significant differences in the antioxidant activity of the new genotypes.

Considering all three antioxidant assays and the ACI values, ‘Egnatia N.’ showed the highest value (95.7%), followed by ‘Appia N.’ (84.96%) and ‘Aika N.’ (82.74%).

### 3.3 Antiproliferative effects on human colon cancer cells

**Figure 2:**
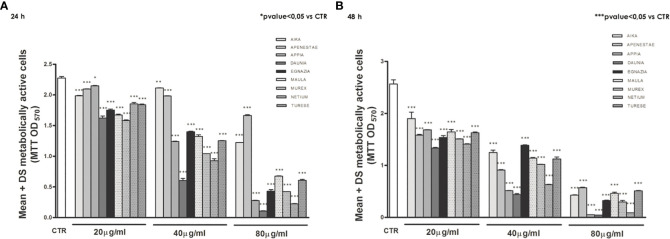
The antiproliferative effects of polyphenols of the nine GSEs on Caco2 cell proliferation. Treated with increasing concentrations (20, 40, 80 μg/mL), both after 24 **(A)** and 48 h **(B)** of treatment. Data were expressed as mean ± standard deviation. p-Value was determined by Paired T-test; * p < 0.05; *** p < 0.001.

The antiproliferative activity of the nine GSEs on Caco2 cell proliferation (**Figure 2**) has been measured by the colorimetric MTT assay. The yellow tetrazolium salt MTT is reduced to purple formazan only by viable cells, which can be spectrophotometrically quantified. Therefore, the coloration of formazan is directly proportional to cell viability.

Exposure of the Caco2 cell line to increasing concentrations (20, 40, 80 μg/mL) of these nine GSEs showed an antiproliferative action of all GSEs starting from a concentration of 20 μg/mL, both after 24 (**Figure 2A**) and 48 h of treatment (**Figure 2B**), revealing that grape phenolic compounds inhibit cell proliferation in a dose- and time-dependent manner.

The antiproliferative activities of different grape skin extracts were also expressed as the median effective dose (EC50), with a lower EC50 value representing a higher antiproliferative activity. The EC50 values of GSEs varied among the new genotypes (**Table 2**). ‘Daunia N.’ showed the highest antiproliferative activity against Caco2 in both experimental times, with the lowest EC50 value of 35.60 µg/mL and 28.61 µg/mL after 24 h and 48 h, respectively. Instead, ‘Apenestae N.’ possessed the lowest antiproliferative activity after 24 h with the EC50 value of 150.03 µg/mL and ‘Aika N.’ after 48 h with EC50 value of 44.49 µg/mL.

**Table 2:** Antiproliferative activities (EC50 after 24 h and EC50 after 48 h) of the nine new grape genotypes toward the Caco2 cell line.

Genotype	EC50_24h	EC50_48h
	(µg/mL)	(µg/mL)
Aika N.	95.90 ± 5.33d	44.49 ± 0.55e
Apenestae N.	150.91 ± 12.91e	40.34 ± 2.21cd
Appia N.	48.07 ± 1.16bc	32.54 ± 1.01b
Daunia N.	35.60 ± 0.67a	28.81 ± 1.19a
Egnatia N.	49.42 ± 1.37bc	41.60 ± 2.30de
Maula N.	53.13 ± 1.96c	41.87 ± 2.68de
Murex N.	43.54 ± 1.16ab	37.46 ± 1.17c
Netium N.	41.93 ± 0.622ab	31.34 ± 1.18ab
Turese N.	52.01 ± 1.25c	42.2 ± 1.62de

In each column, values followed by different letters are statistically different according to the Tukey’s *post hoc* test (*p* < 0.05).

### 3.4 UHPLC/QTOF polyphenolic profiles

**Figure 3:**
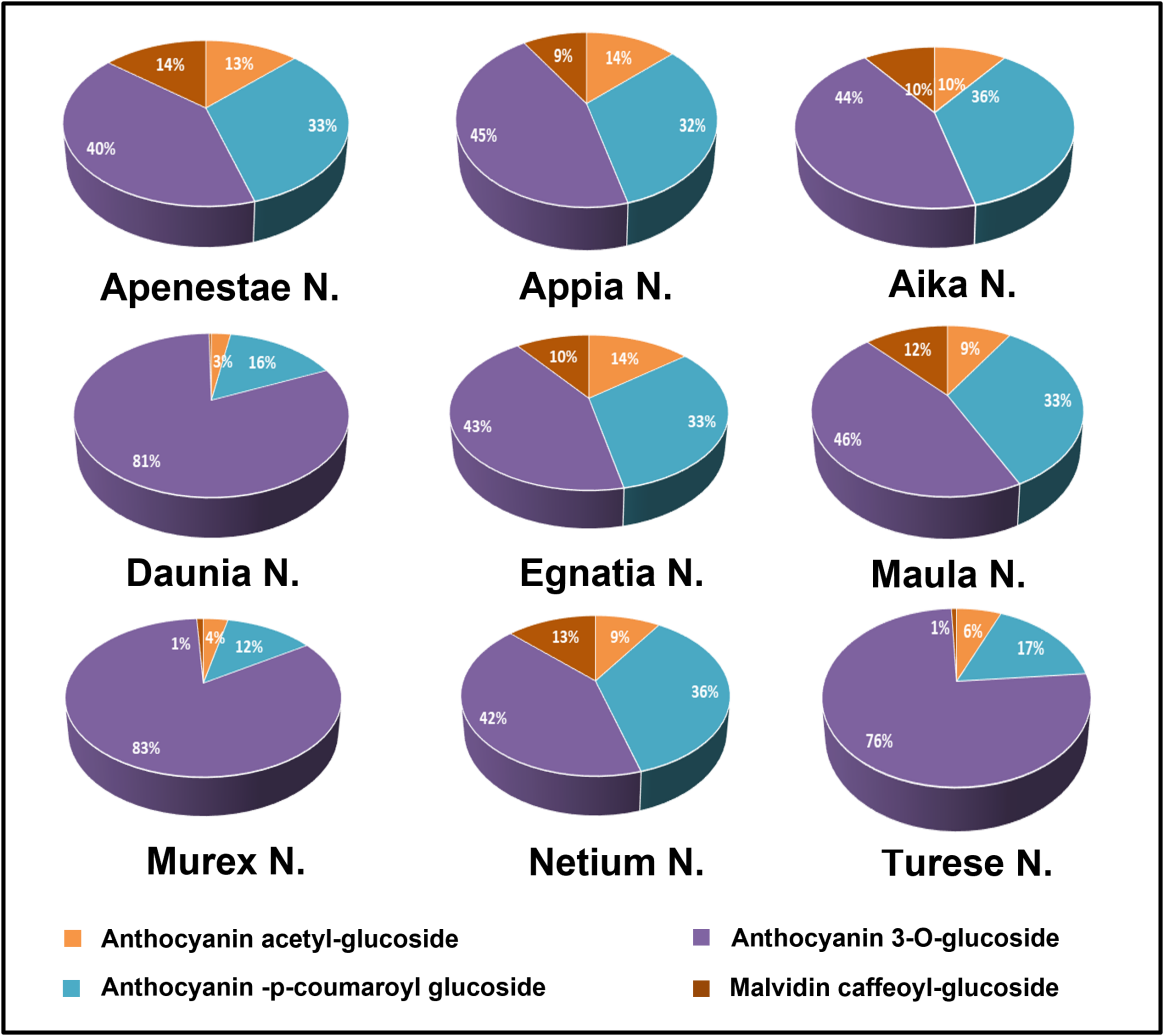
Grape skin anthocyanin composition of the new genotypes: the single classes of compounds were expressed as relative percentages (%) of the sum of M^+^ signal intensities recorded in the positive-ionization UHPLC/QTOF chromatogram.

Anthocyanic compositions of grape skin extracts of the genotypes studied are shown in **Figure 3** where the single classes of anthocyanins are expressed as percentages by summing the M^+^ ions intensity recorded in the positive-ionization mode ultra-high performance liquid chromatography/quadrupole-time of flight (UHPLC/QTOF) chromatogram. Intensities of anthocyanin M^+^ signals recorded in the positive ion chromatogram are reported in **Supplementary Material Table S1**, those of [M-H]^-^ ions of the phenolic compounds recorded in the negative ion chromatogram in **Table S2**. More than one hundred polyphenols including both flavonoid and non-flavonoid compounds were identified in the GSEs. In general, the anthocyanins in *V.vinifera* grapes are delphinidin, cyanidin, petunidin, peonidin, and malvidin present as 3-O-glucoside, 3-O-acetylglucoside and 3-O-p-coumaroylglucoside derivatives. Moreover, malvidin 3-O-caffeoylglucoside is usually present. Anthocyanin profile of all genotypes studied was characterized by glucoside anthocyanins as main compounds, followed by p-coumaroylglucosides then acetylglucosides (**Figure 3**). In particular, the profile of genotypes’ Turese N.’, ‘Daunia N.’, and ‘Murex N.’ had high percentage of non-acylated anthocyanins (75%, 81%, and 83%, respectively) and malvidin-caffeoylglucoside was negligible (around 1%, no found in ‘Daunia N.’), in ‘Aika N.’, ‘Apenestae N.’, ‘Egnatia N.’, ‘Netium N.’, ‘Appia N.’, and ‘Maula N.’ had similar percentages of glucosides (around 43%).

**Figure 4:**
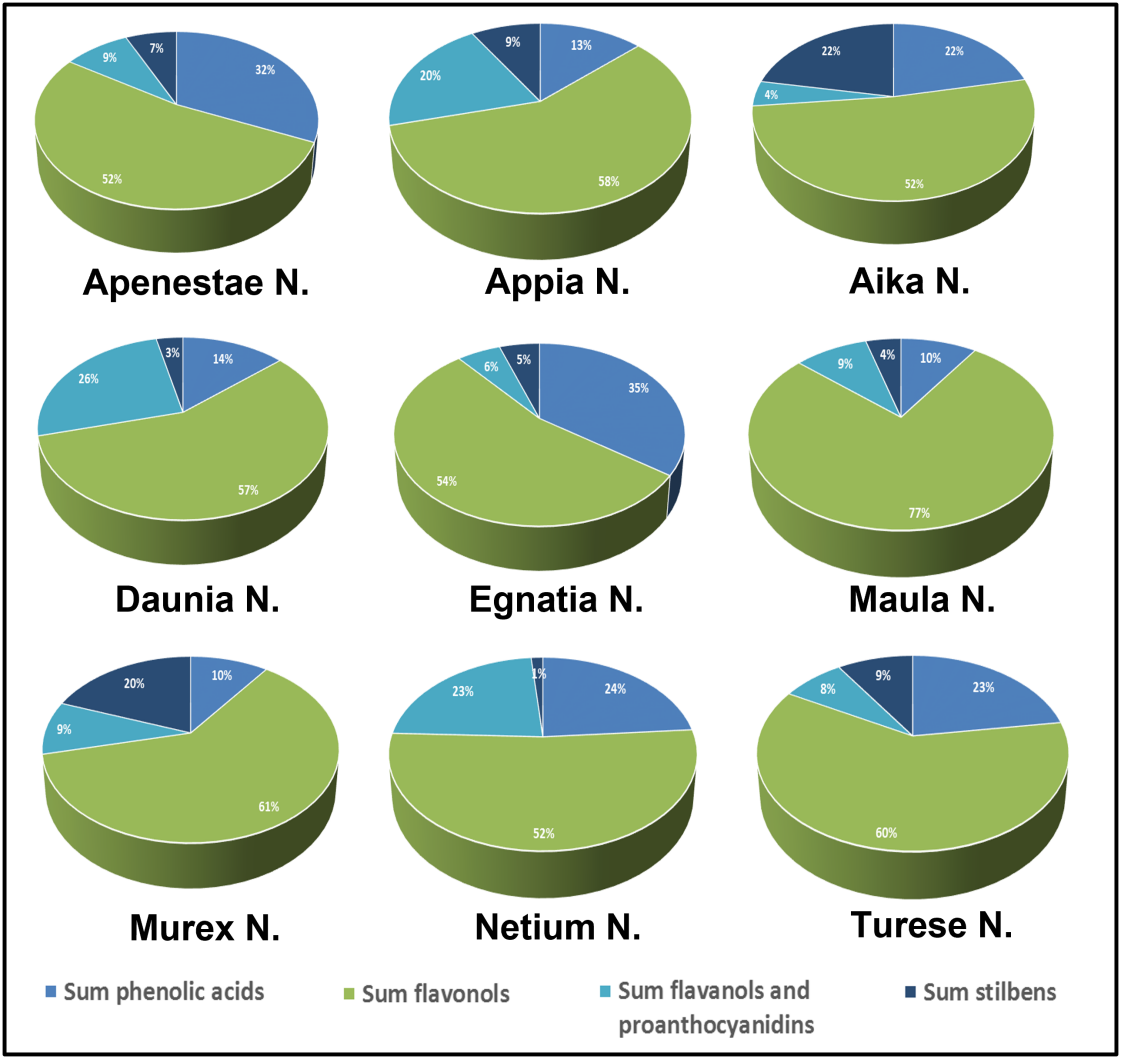
Polyphenolic composition of skin extracts of the new grape genotypes: relative percentages (%) of the single classes of compounds were expressed on the sum of [M-H]^-^ signal intensities recorded in the negative-ionization UHPLC/QTOF chromatogram.

**Figure 4** shows the percentage composition of the different classes of polyphenols calculated by summing the [M-H]^-^ signals intensity recorded in the negative-ion UHPLC/QTOF chromatogram. All the genotypes showed as main signals those of flavonols and flavanonols, which had a total signal intensity percentage between 51-61% of total polyphenols and 76.8% in sample ‘Maula N.’ (**Figure 4**). Higher percentages of flavan-3-ols and proanthocyanidins were found in ‘Daunia N.’, ‘Netium N.’, and ‘Appia N.’ grapes (between 20-25%) while they were lower 10% in the other samples. Relevant variability of phenolic acids in the samples was observed, with percentages ranging between 9-35%. In particular, higher signals were found in ‘Egnatia N.’ and ‘Apenestae N.’ (<30%) followed by ‘Netium N.’ and ‘Aika N.’. Also, the signals of stilbenes were considerably variable, ranging from 1.5% in ‘Netium N.’ to 22.1% in ‘Aika N.’. In a previous study (Tutino et al., 2020b) the catechol percentage was calculated as sum of signal intensities of the o-diphenol derivatives identified in two GSEs. By using this approach, the genotype ‘Turese N.’ has highest catechol percentage (100%) followed by ‘Egnatia N.’ (82%), ‘Murex N.’ (61%), ‘Daunia N.’ (57%), ‘Aika N.’ (47%), and ‘Apenestae N.’, ‘Appia N.’, and ‘Maula N.’ (27%).

### 3.5 Correlation between phenolics, antioxidant properties, and antiproliferative activities

The correlations between phenolic contents, antioxidant properties, and antiproliferative activities are summarized in **Table 3**. A strong correlation was found between the antioxidant activity (ABTS and ORAC) and TPC, TF, and TA. Only TPC had significantly positive correlations with the DPPH antioxidant activity. Data also showed correlations between the signal intensity of the metabolite groups identified in the UHPLC/QTOF chromatogram and GSEs’ antioxidant and antiproliferative activity. Correlation of anthocyanin glucosides, anthocyanin acetylglucosides, phenolic acids, and stilbenes to the antioxidant assays, was found; instead, no correlation was observed between antioxidant activity and flavanol/procyanidin and flavonol signals.

**Table 3:** Pearson’s correlation coefficients of total phenolic content (TPC), total anthocyanins (TA) and total flavonoids (TF), phenolic compound groups (anthocyanins, flavanol/proanthocyanidins, flavonols, phenolic acids, and stilbenes), antioxidant activity (ABTS, DPPH, ORAC) and median concentration of antiproliferative activity (EC50 after 24h and EC50 after 48h).

	ABTS	DPPH	ORAC	EC50_24h	EC50_48h
	(µM TE/g)	(µM TE/g)	(µM TE/g)	(µg/mL)	(µg/mL)
TPC	0.894	0.622	0.776	–	–
TF	0.747	–	0.665	–	–
TA	0.707	–	0.738	–	0.692
ABTS	1	0.5	0.834	–	–
DPPH	0.5	1	0.402	–	–
ORAC	0.834	0.402	1	0.442	0.399
sum of anthocyanins	0.544	0.475	0.484	0.382	0.411
- 3-*O*-glucoside	0.537	0.475	0.634	–	0.482
- *p*-coumaroylglucoside	0.449	–	–	–	–
- acetylglucoside	0.591	0.589	0.479	0.402	–
- malvidin-caffeoylglucoside	–	–	–	0.518	–
flavanols/proanthocyanidins sum	–	–	–	-0.419	-0.922
sum of flavonols	–	–	–	–	–
sum of phenolic acids	0.429	0.447	0.322	–	–
sum of stilbenes	0.461	–	0.428	–	0.503
sum of *o*-diphenol compounds	–	0.387	0.445	–	–

The correlation was considered very strong when r = 0.90-0.99 (positive) or -0.99-(-0.99) (negative); strong when r = 0.70-0.89 (positive) or -0.89-(-0.70) (negative); and moderate when r = 0.40-0.69 (positive) or -0.69-(-0.40) (negative).

No significant correlation was found between total phenolic contents and the antiproliferative activities, except for TA showing a moderate positive correlation with the EC50 after 24 h (r = 0.692). Interestingly, a negative correlation was found between flavanols/proanthocyanidins and antiproliferative activity, moderate after 24h (r = -0.419) and strong after 48h (r = 0.922). No significant correlations were found between antioxidant assays and the antiproliferative activities except for ORAC values which showed a moderate positive correlation with EC50 after 24h (r = 0.442). Thus, the correlations between individual compounds of each group of phenolics and antioxidant and antiproliferative activity were investigated. The complete data are reported in the **Supplementary Table S3**, while **Table 4** shows the correlation between flavanols/proanthocyanidins and antioxidants and the antiproliferative activities. A very strong negative correlation was found between procyanidin T2, T3, C1_A, epigallocatechin, and antiproliferative activity at 48h.

**Table 4:** Pearson’s correlation coefficients of flavan-3-ols/procyanidins, antioxidant activity (ABTS, DPPH, ORAC) and median concentration of antiproliferative activity (EC50 after 24h and EC50 after 48h).

	ABTS	DPPH	ORAC	EC50_24h	EC50_48h
	(µM TE/g)	(µM TE/g)	(µM TE/g)	(µg/mL)	(µg/mL)
Flavan-3-ols/procyanidins					
(-)-epicatechin	-0.532	-0.531	-0.504	–	-0.585
(+)-catechin	–	-0.466	-0.395	–	–
(epi)gallocatechin isomer	–	–	–	–	-0.66
(-)-epicatechin gallate	–	–	–	-0.385	-0.851
procyanidin (B3/B4/B5)	–	–	–	–	-0.8383
procyanidin B1	–	–	–	-0.408	-0.897
procyanidin B2	–	–	–	-0.456	-0.856
(epi)gallocatechin-catechin 1	–	–	–	-0.394	-796
(epi)gallocatechin-catechin 2	–	–	–	-0.474	-0.93
procyanidin T2/T3(T4)/C1 1	–	–	–	-432	-0.903
procyanidin T2/T3(T4)/C1 2	–	–	–	-0.441	-0.885
procyanidin T2/T3(T4)/C1 3	–	–	–	-0.503	-0.731
prodelphinidin T2/T3 1	–	–	–	-0.445	-0.89
prodelphinidin T2/T3 2	–	–	–	-0.472	-0.859
prodelphinidin T1	–	–	–	-0.34	-0.619

## 4 Discussion

Over time, consumer needs and expectations have been changing with a growing awareness of the importance of the nutraceutical properties of fruit and vegetables, promoting well-being and reducing the risk of developing diseases. The association between diet, health, and bioactive compounds in food is evident. For this reason, “functional foods” containing components that can protect our health are becoming very popular. Grape is qualitatively and quantitatively rich in bioactive compounds such as polyphenols, and as suggested by our previous studies, the intake of fresh table grapes induces health benefits (Ammollo et al., 2017; Milella et al., 2020; Tutino et al., 2021).

Polyphenolic composition is closely linked to the genotype and can differ considerably among the grape varieties. The table grape breeding program of CREA started eighteen years ago and produced thousands of new plants. Thirty-six of them in 2018 were selected by a consortium of Apulian companies, NUVAUT (NUove VArietà Uve da Tavola), intending to exploit them commercially. Therefore, for eleven of these thirty-six selections, the European Patent at the Community Plant Variety Office (CPVO) was obtained. Furthermore, for three of these, registration at the National Catalog for vine varieties was requested at the Ministry for Agricultural Food and Forestry Policies. Nine of these thirty-six new table grape genotypes were selected to investigate their GSEs’ antioxidant and anticancer activity.

The most interesting biological function of phenolic compounds is to maintain oxidative stress levels below a critical point in the body. This suppression of oxidants occurs by donating electrons or a hydrogen atom from the free hydroxyls or chelating metal ions, thus preventing radical-induced damage in biological systems. Combining the results from all performed tests (DPPH, ABTS, and ORAC), the ‘Egnatia N.’ reported the highest antioxidant activity among all new genotypes, followed by ‘Appia N.’ and ‘Aika N.’. Moreover, the positive correlation among TPC, TF, TA, and antioxidant activity was in agreement with what was reported by Liang et al. (2014) and Xia et al. (2019).

The UHPLC/QTOF investigation provided a complete picture of the bioactive compounds considering that only a few studies have investigated the polyphenolic composition of table grapes. This detailed characterization also allowed us to evaluate the correlations between the polyphenol composition of GSEs and their antioxidant and antiproliferative activity.

In general, grape polyphenols are formed by many ortho-diphenol structures, which determine the antioxidant activity of the extract. Monophenols are less reducer than diphenols and triphenols because o-position establishes an intramolecular H-bond between the hydroxyl groups by stabilizing the phenoxy free radical and enhancing the reducing properties of the molecule (Spiegel et al, 2020). Moreover, larger delocalization of the unpaired electron resulting from the antioxidative action enhances the reducing properties of the phenol derivatives: for instance, caffeic acid is a better reducer than protocatechuic acid, and quercetin than catechin or epicatechin (Laguerre et al., 2014).

The first class of compounds analyzed was anthocyanins which have many biological functions in plants, such as protection against UV radiation, pathogen attacks, oxidative damage, and attraction of animals for seed dispersal. In grapes, anthocyanins are present as 3-O-glucoside, acetylmonoglucoside, and p-coumaroylmonoglucoside derivatives and are responsible for fruit color. In our study, ‘Daunia N.’, ‘Murex N.’, and ‘Turese N.’ differ significantly from other genotypes because they have almost double 3-O-glucoside derivatives and percentage of delphinidin petunidin, and malvidin derivatives are lower than others. This result could be explained by the fact that, as described above, these three genotypes derived from a single crossing (‘Red Globe RS.’ x ‘Regal B.’), confirming that grape anthocyanin composition is closely linked to the genetic profile and can differ considerably among the varieties, so it is also studied for chemotaxonomic purposes.Considering the anthocyanin’s chemical structure and the hydroxyl group’s role in increasing the antioxidant activity (Mattioli et al., 2020), the correlation between anthocyanin composition and antioxidant activity was investigated. The 3-O-glucoside and acetylglucoside anthocyanin groups correlated moderately with the three antioxidant assays. Interestingly, delphinidin and petunidin derivatives seemed to be involved in the antioxidant activity, contrary to cyanidin and malvidin derivates. Specifically, delphinidin and petunidin acetylglucoside were highly correlated with the antioxidant tests. Our findings agree with the knowledge that the free radical scavenging effect enhances by increasing the number of the hydroxyl group in the flavonoid, considering that delphinidin and petunidin are derivatives trihydroxylated at the B-ring.

The new grape genotypes also showed different profiles of non-anthocyanin flavonoids: ‘Maula N.’ was the richest in flavonols, ‘Apenestae N.’ in phenolic acids, ‘Daunia N.’ in flavanols and proanthocyanidins, ‘Aika N.’ in stilbenes. The Pearson coefficient analysis showed a moderate correlation between phenolic acids and stilbenes and antioxidant activity among the analyzed polyphenolic classes. Phenolic acids are known as direct antioxidants due to the reactivity of phenol moiety with a great radical scavenging activity *via* hydrogen atom donation (Kumar and Goel, 2019). Among the phenolic acids identified in our study, gallic acid glucoside and syringic acid glucoside highly correlated with the three antioxidant assays. Stilbenes are phytoalexins partially influenced by genetics and are produced to protect the plant from external factors such as biotic and abiotic stresses which affect their levels in grapes. (Bavaresco et al., 2012). A moderate correlation was observed between viniferins (E and Z ε-viniferins, Z-ω-viniferin, and δ-viniferin) and GSEs antioxidant activity.

The antiproliferative activity of GSEs on human colon cancer cells was investigated. Many studies reported the potential of polyphenols as therapeutic agents against several types of cancer, defining them as potential antiproliferative agents. They can exert multiple health benefits by acting as prooxidants with the cumulative production of ROS and oxidative damage to membrane lipids and other cellular constituents to cause cancer cell death (Arau´jo Vieira do Carmo et al., 2018). Emerging evidence highlights the numerous anticarcinogenic mechanisms of flavonoids, including proliferation inhibition, the induction of apoptosis, autophagy, necrosis, cell cycle arrest, senescence, the impairment of cell migration, invasion, tumor angiogenesis, and the reduction of multidrug resistance in tumor cells. Valenzuela et al. (2018) reported chemopreventive and anticancer properties of grape juice extracts from ‘Autumn Royal N.’ and ‘Ribier N.’ varieties which inhibit the growth of HT-29 and SW-480 colon cancer cells in a dose-dependent manner. Gigante et al. (2020), investigated the effects of grape skin extracts from ‘Autumn Royal N.’ and ‘Egnatia N.’ on Caco2, HT29 and SW480 human colon cancer cell lines. Even if the total polyphenolic content and the total antioxidant capacity were significantly higher in ‘Autumn Royal N.’ than in ‘Egnatia N.’, table grape ‘Egnatia N.’ showed greater ability to affect cell proliferation and apoptosis, as well as to exert a growth arrest in the S phase of the cell cycle, particularly in the Caco2 cell line (Gigante et al., 2020). In our study, all genotypes inhibited the proliferation of Caco2 cells, with ‘Daunia N.’, ‘Netium N.’, and ‘Appia N.’ having the highest antiproliferative effects on cancer cells.

Anthocyanins are known for their protective action on cancer (Thilavech et al., 2018; Medic et al., 2019; Saulite et al., 2019). In our experimental conditions, the anthocyanins seem not to contribute to the antiproliferative activity, reporting a moderate positive correlation coefficient between anthocyanin 3-O-glucoside and EC50, indicating their non-involvement in the anticancer activity of GSEs. The lack of correlation between anthocyanins and antiproliferative activity observed in our experimental condition does not agree with other literature findings (Urias-Lugo et al., 2015; Lin et al., 2017). On the contrary, a strong correlation of flavanols/proanthocyanidins with the antiproliferative activity of GSEs was found. Flavan-3-ols, called flavanols here, are an important subclass of flavonoids. They are present in grape as monomers or polymers called proanthocyanidins or condensed tannins. In skins and seeds, four principal units of PAs are usually found: (+)-catechin, (−)-epicatechin, (−)-epigallocatechin and (−)-epicatechin gallate. Grape seeds contain greater amounts of (−)-epicatechin gallate and all flavanols. Proanthocyanidins polymers are longer in skins than seeds (Zerbib et al., 2018). Literature on health aspects of proanthocyanidins reports their anticarcinogenic activities and capacity to interfere with multiple pathways associated with the growth and progression of various cancer, such as breast cancer, colorectal and prostatic cancers (Rauf et al., 2019; Wang et al., 2020). Our study revealed a high correlation between epigallocatechin and a procyanidin trimer with the antiproliferative activity of GSEs.

In conclusion, the nine new table grape genotypes showed engaging nutritional quality. Findings revealed that several classes of compounds, such as anthocyanins, phenolic acids, and stilbenes, contribute to the GSE antioxidant activity, suggesting a synergic effect of these compounds. On the other hand, specific compounds, flavanols and proanthocyanidins, resulted in major drivers of antiproliferative activities in complex polyphenol mixtures from grape skin extracts. These results are consistent with those reported in the literature (Kallithraka et al., 2009; Liang et al., 2014) and increase the knowledge of the health effects of grape polyphenols.

This investigation may lead to further studies *in vitro* and *in vivo* to support the antioxidant and antiproliferative effects. It is crucial to consider that the potential activities of phenolic compounds may be limited by their bioavailability and metabolism in the gut and liver. The biological activities of phenolic compounds may be mediated by their metabolites produced *in vivo.* Several factors must be considered, such as interaction with the food matrix, the metabolic processes mediated by the liver (phase I and II metabolism), the intestine, and microbiota (Di Lorenzo et al., 2021). Studies in which the extracts undergo *in vitro* digestion are the first step in evaluating bioavailability. However, this work provided a better understanding of the structure-activity relationship of phenolic compounds that could assist grape geneticists in breeding programs, producers in cultivating grape varieties with more significant health benefits, consumers in selecting grape varieties with high nutraceutical value, and scientists in developing new anticancer therapies.

## Data availability statement

The original contributions presented in the study are included in the article/**Supplementary Material**. Further inquiries can be directed to the corresponding authors.

## Author contributions

RM planed and conceptualized the manuscript; VT, MR, IG, LF, AM, and RP performed the experiments; GD analyzed the data; MG and MN discussed the data; RM wrote the manuscript; RV and RF revised the manuscript. All authors contributed to the article and approved the submitted version.
