# Corrigendum: Correlation between antioxidant and anticancer activity and phenolic profile of new Apulian table grape genotypes (*V. Vinifera* L.)

**DOI:** 10.3389/fpls.2023.1187227

**Published:** 2023-03-24

**Authors:** Rosa Anna Milella, Mirko De Rosso, Marica Gasparro, Isabella Gigante, Giambattista Debiase, Lucia Rosaria Forleo, Antonio Domenico Marsico, Rocco Perniola, Valeria Tutino, Maria Notarnicola, Riccardo Velasco, Riccardo Flamini

**Affiliations:** ^1^ Research Centre for Viticulture and Enology, Council for Agricultural Research and Economics, Turi, Bari, Italy; ^2^ Research Centre for Viticulture and Enology, Council for Agricultural Research and Economics, Conegliano, Treviso, Italy; ^3^ Laboratory of Nutritional Biochemistry, National Institute of Gastroenterology Saverio de Bellis, IRCCS Research Hospital, Castellana Grotte, Italy

**Keywords:** breeding, phenolic compounds, seedless grape, antioxidant, anticancer, quality improvement

In the published article, there was an error in affiliation 3. Instead of “Laboratory of Nutritional Biochemistry, National Institute of Gastroenterology “S. de Bellis” Research Hospital, Castellana Grotte (BA), Italy”, it should be “Laboratory of Nutritional Biochemistry, National Institute of Gastroenterology Saverio de Bellis, IRCCS Research Hospital, Castellana Grotte, Italy”.

The authors apologize for this error and state that this does not change the scientific conclusions of the article in any way. The original article has been updated.

