# Supplementary material for: Correlation between antioxidant and anticancer activity and phenolic profile of new Apulian table grape genotypes (V. Vinifera L.)
Source: Front Plant Sci. 2023 Jan 11;13:1064023. doi: 10.3389/fpls.2022.1064023 (PMC9874228; doi:10.3389/fpls.2022.1064023)
Supplement: Supplementary file 3 [file Table_3.docx]

**Table S3** Pearson’s correlation coefficients of phenolic compounds, antioxidant activity (ABTS, DPPH, ORAC) and median concentration of antiproliferative activity (EC50 after 24h and EC50 after 48h).

|  |  | **ABTS**  **(µM TE/g)** | **DPPH**  **(µM TE/g)** | **ORAC**  **(µM TE/g)** | **EC50_24h**  **(µg/mL)** | **EC50_48h**  **(µg/mL)** |
| --- | --- | --- | --- | --- | --- | --- |
| **Anthocyanins** |  |  |  |  |  |  |
| cyanidin-acetylglucoside |  | - | - | - | - | - |
| cyanidin-p-coumaroylglucoside |  | - | - | - | - | - |
| cyanidin-3-O-glucoside |  | - | - | - | - | - |
| delphinidin-acetylglucoside |  | 0.498 | 0.893 | - | - | - |
| delphinidin-p-coumaroylgluc. |  | 0.457 | 0.596 | - | - | - |
| delphinidin-3-O-glucoside |  | - | 0.680 | - | - | - |
| malvidin-acetylglucoside |  | 0.515 | 0.537 | 0.387 | - | - |
| malvidin caffeoylglucoside |  | - | - | - | 0.518 | - |
| malvidin-p-coumaroylglucoside |  | - | - | - | 0.398 | - |
| malvidin-3-O-glucoside |  | - | - | - | - | - |
| peonidin-acetylglucoside |  | - | - | 0.458 | - | 0.389 |
| peonidin-p-coumaroylglucoside |  | 0.441 | - | 0.475 | - | 0.399 |
| peonidin-3-O-glucoside |  | - | - | - | - | - |
| petunidin-acetylglucoside |  | 0.579 | 0.813 | 0.450 | - | - |
| petunidin-p-coumaroylglucoside |  | 0.544 | - | 0.493 | - | - |
| petunidin-3-O-glucoside |  | 0.608 | 0.777 | 0.465 | - | 0.421 |
| **Phenolic acids** |  |  |  |  |  |  |
| shikimic acid |  | 0.464 | - | 0.414 | - | - |
| methylgallic acid isomer 1 |  | 0.420 | - | - | - | - |
| methylgallic acid isomer 2 |  | - | - | - | - | - |
| 5-hydroxy-ferulic acid |  | - | - | - | - | - |
| hydroxybenzoic acid glucoside |  | - | 0.579 | - | - | - |
| dihydroxybenzoic acid glucoside |  | - | - | - | - | - |
| feruloyltartaric acid |  | - | - | - | - | - |
| vanillic acid glucoside |  | - | 0.466 | - | - | - |
| gallic acid glucoside |  | 0.484 | 0.454 | 0.484 | 0.503 | - |
| ferulic acid glucoside |  | - | - | - | - | - |
| syringic acid glucoside |  | 0.575 | 0.406 | 0.495 | 0.540 | -0.568 |
| cis-p-coumaroyltartaric acid |  | -0.428 | - | - | - | - |
| trans-p-coumaroyltartaric acid |  | - | - | - | -0.427 | - |
| trans-caffeoyltartaric acid |  | - | - | - | - | - |
| **Flavonol and flavanonols** |  |  |  |  |  |  |
| kaempferol |  | -0.575 | -0.507 | -0.427 | -0.508 | -0.382 |
| quercetin |  | -0.460 | -0.414 | - | - | - |
| tamarixetin/isorhamnetin |  | - | - | - | - | - |
| myricetin |  | 0.675 | 0.541 | 0.464 | - | - |
| syringetin |  | 0.460 | 0.580 | - | - | - |
| quercetin-pentoside |  | - | - | - | - | -0.429 |
| taxifolin-pentoside |  | - | - | - | - | -0.645 |
| kaempferol-3-O-galactoside |  | -0.407 | - | - | -0.499 | - |
| kaempferol-3-O-glucoside |  | - | -0.390 | - | - | - |
| dihydroquercetin-rhamnoside |  | - | - | - | - | -0.666 |
| tetrahydroxyflavanone-hex. 1 |  | - | -0.454 | - | - | - |
| tetrahydroxyflavanone-hex. 2 |  | - | - | - | - | - |
| kaempferol-3-O-glucuronide |  | - | - | - | - | - |
| quercetin-3-O-galactoside |  | - | - | - | - | -0.431 |
| quercetin-3-O-glucoside |  | - | - | - | - | - |
| quercetin-3-O-glucuronide |  | -0.713 | -0.612 | -0.588 | 0.498 | -0.627 |
| isorhamnetin-3-O-glucoside |  | - | - | - | - | - |
| myricetin-3-O-glucoside |  | 0.634 | 0.490 | 0.451 | - | - |
| myricetin-3-O-glucuronide |  | 0.432 | - | - | - | - |
| laricitrin-3-O-glucoside |  | 0.455 | - | - | - | - |
| laricitrin-glucuronide |  | - | - | - | - | - |
| syringetin-3-O-glucoside |  | - | - | - | - | - |
| tetrahydroxy-dimethoxyflavanone-hexoside |  | - | - | - | - | - |
| rutin (quercetin-3-O-rutinoside) |  | - | - | - | - | - |
| Isorhamnetin-p-coumaroylgluc. |  | 0.581 | 0.4815 | 0.493 | 0.536 | - |
| quercetin-diglucoside |  | -0.532 | -0.504 | -0.531 | - | -0.585 |
| dihydroquercetin-O-hexoside 1 |  | 0.513 | - | 0.432 | - | - |
| dihydroquercetin-O-hexoside 2 |  | - | - | - | - | - |
| **Stilbenes** |  |  |  |  |  |  |
| *trans*-resveratrol |  | - | - | - | - | - |
| piceatannol |  | - | - | - | 0.441 | - |
| *cis*-piceid |  | - | - | - | - | 0.387 |
| *trans*-piceid |  | - | - | - | - | 0.481 |
| E-astringin |  | - | - | - | - | 0.492 |
| Z-astringin |  | - | - | - | - | 0.414 |
| pallidol |  | - | - | - | - | 0.552 |
| resveratrol dimer |  | - | - | 0.410 | - | - |
| Z-e-viniferin |  | 0.544 | - | - | - | - |
| Z-ω-viniferin |  | 0.642 | - | 0.520 | - | - |
| E-ε-viniferin |  | 0.545 | - | 0.399 | - | - |
| δ-viniferin |  | 0.430 | - | - | 0.503 | 0.549 |
| pallidol-O-glucoside |  | - | - | - | - | 0.475 |
| α-viniferin |  | - | - | - | - | 0.442 |
| Z-miyabenol C |  | 0.415 | - | - | - | 0.510 |
| E-miyabenol C |  | - | - | - | - | 0.480 |
| resveratrol tetramer 1 |  | - | - | - | - | 0.504 |
| resveratrol tetramer 2 |  | - | - | - | - | 0.406 |
|  |  |  |  |  |  |  |
|  |  |  |  |  |  |  |
|  |  |  |  |  |  |  |
